# Peptidomic investigation of *Neoponera villosa* venom by high-resolution mass spectrometry: seasonal and nesting habitat variations

**DOI:** 10.1186/s40409-018-0141-3

**Published:** 2018-02-17

**Authors:** Camila Takeno Cologna, Renata Santos Rodrigues, Jean Santos, Edwin de Pauw, Eliane Candiani Arantes, Loïc Quinton

**Affiliations:** 1 School of Pharmaceutical Sciences of Ribeirão Preto, University of São Paulo, Ribeirão Preto, SP Brazil; 2 Laboratory of Mass Spectrometry, MolSys, Department of Chemistry, Liège Université, Liège, Belgium; 3 Federal University of Uberlândia, Uberlândia, MG Brazil

**Keywords:** Ant venom, Peptidomics, Ponericins, Venom comparison, Antimicrobial peptides

## Abstract

**Background:**

Advancements in proteomics, including the technological improvement in instrumentation, have turned mass spectrometry into an indispensable tool in the study of venoms and toxins. In addition, the advance of nanoscale liquid chromatography coupled to nanoelectrospray mass spectrometry allows, due to its high sensitivity, the study of venoms from species previously left aside, such as ants. Ant venoms are a complex mixture of compounds used for defense, predation or communication purposes. The venom from *Neoponera* ants, a genus restricted to Neotropical regions, is known to have cytolytic, hemolytic, antimicrobial and insecticidal activities. Moreover, venoms from several *Neoponera* species have been compared and differences in their toxicity related to nesting habitat variation were reported. Therefore, the present study aimed to perform a deep peptidomic analysis of *Neoponera villosa* venom and a comparison of seasonal and nesting habitat variations using high-resolution mass spectrometry.

**Methods:**

Specimens of *N. villosa* ants were captured in Panga Natural Reserve (Uberlândia, MG, Brazil) from arboreal and ground-dwelling nests during summer and winter time. The venom glands were dissected, pooled and disrupted by ultra-sonic waves. The venom collected from different habitats (arboreal and ground-dwelling) and different seasons (summer and winter) was injected into a nanoACQUITY ULPC hyphened to a Q-Exactive Orbitrap mass spectrometer. The raw data were analyzed using PEAKS 7.

**Results:**

The results showed a molecular diversity of more than 500 peptides among these venoms, mostly in the mass range of 800–4000 Da. Mutations and post-translational modifications were described and differences among the venoms were observed. Part of the peptides matched with ponericins, a well-known antimicrobial peptide family. In addition, smaller fragments related to ponericins were also identified, suggesting that this class of antimicrobial peptide might undergo enzymatic cleavages.

**Conclusion:**

There are substantial differences among the venom of *N. villosa* ants collected in different seasons and from different nest habitats. The venom composition is affected by climate changes that influence prey availability and predator presence. Clearly, nano-LC-MS boosted the knowledge about ant venom, a rich source of unexplored and promising bioactive compounds.

**Electronic supplementary material:**

The online version of this article (10.1186/s40409-018-0141-3) contains supplementary material, which is available to authorized users.

## Background

Unquestionably, fundamental research on Hymenoptera venom benefits a great deal from the development of miniaturized peptidomics and the improvements in nanoscale liquid chromatography coupled to nanoscale tandem mass spectrometry (nano-LC-MS/MS). Mostly due to their small size and therefore scarcely collected venom, this order has always been neglected and considered unfeasible to be studied through the known classical strategies that generally involve large amounts of venom [1, 2]. Hymenopterans (sawflies, wasps, ants, and bees) are recognized as one of the most diverse insect order, comprising more than 153,000 described species [3]. Among those, 9100 species correspond to stinging ants, the most abundant group of venomous animals on Earth and ubiquitous in terrestrial environments [4, 5].

Ant venoms vary considerably, but they are generally composed of a complex mixture of peptides and proteins, biogenic amines, hydrocarbons, formic acid and alkaloids [5–7]. This mixture is responsible for a large range of activities including antimicrobial, hemolytic, cytolytic, paralytic, insecticidal and pain inflicting effects [5, 8, 9]. Thus, it can be exploited for different purposes such as defense (against predators, competitors and microbial pathogens), predation and social communication [5, 9, 10]. The dazzling diversity of ant venom composition and function could be a reflection of their preference for different nesting habitats, and consequently their diet and hunting behaviors [2, 5, 10, 11]. This still unexplored extant chemical diversity represents a source of novel bioactive toxins that could be used as tools for the development of new biopesticides and therapeutic agents such as antimicrobials drugs [12].

*Neoponera* genus represents a large group of ants belonging to the Ponerinae subfamily and to date it has 57 described species [13]. The venom from *Neoponera* ants, besides inflicting a painful sting, is known to have cytolytic, hemolytic and antimicrobial activities. In insects, the venom causes paralysis and death, highlighting its bio-insecticidal potential [8, 14]. In addition, the venoms of several *Neoponera* species were compared and the authors observed differences in the toxicity of the venom from ants with arboreal and ground-dwelling nest habitats [10].

In the light of the aforementioned, this study performed a deep peptidomic comparison of *Neoponera villosa* venoms extracted in summer and winter, and from arboreal and ground-dwelling nests through high resolution mass spectrometry, de novo sequencing and in silico identification of peptides.

## Methods

### Venom collection

Specimens of *Neoponera villosa* ant were collected at the Panga Natural Reserve located 30 km south of Uberlândia, Minas Gerais State, Brazil (19° 10′ S, 48° 24′ W) and immediately taken to the laboratory. Arboreal ants and ground-dwelling ants were kept separately. The ants were collected in different seasons (summer and winter) and from two different arboreal nests and one ground-dwelling nest. The venom sacs were dissected, pooled in 15% acetonitrile (ACN) and 0.1% of TFA and disrupted by ultrasonic waves. The empty reservoirs and membrane debris were discarded by centrifugation [8]. The number of venom sacs/condition were: winter (49 sacs); summer (40 sacs); arboreal (30 sacs); ground-dwelling (23 sacs). Total protein quantification was performed for each sample using Bradford assay. The samples were lyophilized and kept at − 20 °C.

### Mass spectrometry approaches

#### Nano-liquid chromatography coupled to electrospray tandem mass spectrometry (Nano-LC-ESI-MS/MS) Orbitrap (Q-Exactive)

Top-down venomics of *N. villosa* venom was selected for exploring and comparing the peptidomes of the venoms collected in different conditions. *N. villosa* crude venoms were diluted in 10 μL 0.2% of FA (formic acid) and injected into a nanoACQUITY ULPC (Waters, UK) hyphened to a Q-Exactive Orbitrap mass spectrometer (Thermo Scientific, USA). The chromatographic system 2D nanoACQUITY ULPC (Waters, UK) was equipped with a monolithic PepSwift Capillary column (100 μm × 25 cm, Thermo Scientific, USA) equilibrated with solution A (H_2_O/0.1% formic acid). The elution of the peptides of each venom condition was performed with a gradient of 3–50% of solution B in 97 min (A: H_2_O/FA 0.1%; B: ACN) at a flow rate of 1 μL/min. All mass spectrometry analyses were performed in data-dependent acquisition (DDA) mode that automatically triggers the MS/MS experiments. The mass range of the mass spectrometry (MS) experiments were set to 400–1750 m/z. The top 12 most intense peaks of each MS scan were fragmented by high-energy dissociation (HCD) and their corresponding MS/MS spectra were acquired in the Orbitrap analyzer on the mass range of 200–2000 m/z. The automatic gain control (AGC) target values were 3e6 for MS spectra and 1e5 for MS/MS spectra.

### Data processing and data base search

Full spectra were deconvoluted and processed using Thermo Scientific Xtract software in order to obtain a peptide mass list. The generated lists of each venom were manually processed and all masses below 800 Da and intensities lower than 1E4 were deleted. The lists were cleaned up by deleting the redundant masses as well. After cleaning the data, we could compare the masses present on the venom of winter and summer, ground-dwelling and arboreal ants.

For each pair of venoms compared (winter × summer; ground-dwelling × arboreal), the masses within ±0.05 Da difference were considered as “common mass”. In parallel, raw data obtained from the Orbitrap analysis were examined in Peaks 7 software (Bioinformatics solutions, Canada). De novo sequencing was performed using a mass tolerance of 10 ppm and mass accuracy of 0.1 Da for the parent and fragment ions, respectively. Oxidation (H, W and M) and amidation were set as variable modifications. The average local confidence (ALC) was established in > 70%. A decoy database was used to calculate the false discovery rate (FDR) which was set to < 1%. The search was carried out against Hymenoptera and animal toxins databases. In order to increase the number of sequences, the Spider algorithm from Peaks software was used. This algorithm explores the best similarity between the de novo sequences determined by proteomics experiments (based on MS/MS spectra) and sequences stored in databases.

## Results

### Venom comparisons – summer × winter

**Fig. 1:**
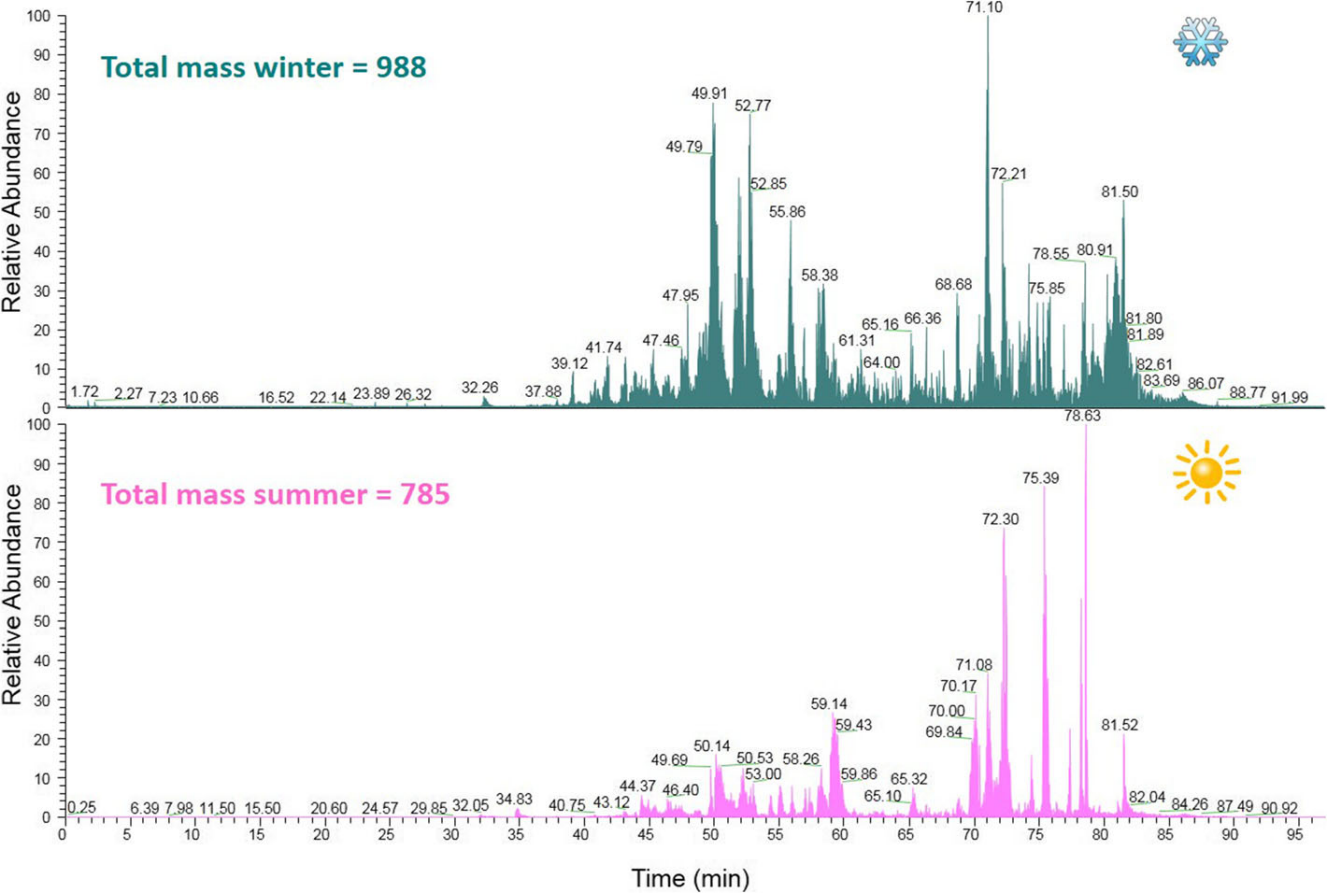
Total ion chromatogram (TIC) of ant venoms extracted during the winter (**blue turquoise**) and summer (**pink**)

**Fig. 2:**
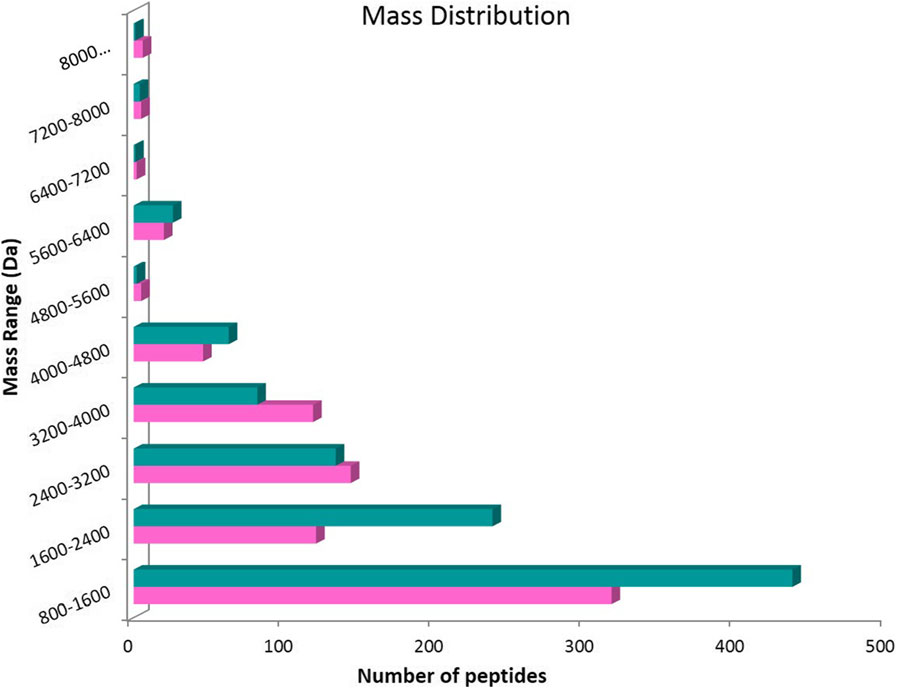
Mass distribution of venom peptides extracted during summer (**pink**) and winter (**blue turquoise**)

The venoms collected during winter and summer were compared using liquid chromatography– mass spectrometry (both venoms were collected from arboreal ants). Most of the peptides were eluted between 35 and 80 min that correspond to 30% of acetonitrile, as shown on the total ion chromatogram (Fig. 1). The spectra were deconvoluted using Xtract and a mass list was generated together with the estimation of the number of peptides present in the venom. A total of 988 peptides between 800 and 10,000 Da were observed in the winter venom, while the summer venom presented 785 different peptides on the same mass range. The mass distribution of the peptides (Fig. 2) was similar, with the majority of the peptides in the range 800–1600 Da.

**Fig. 3:**
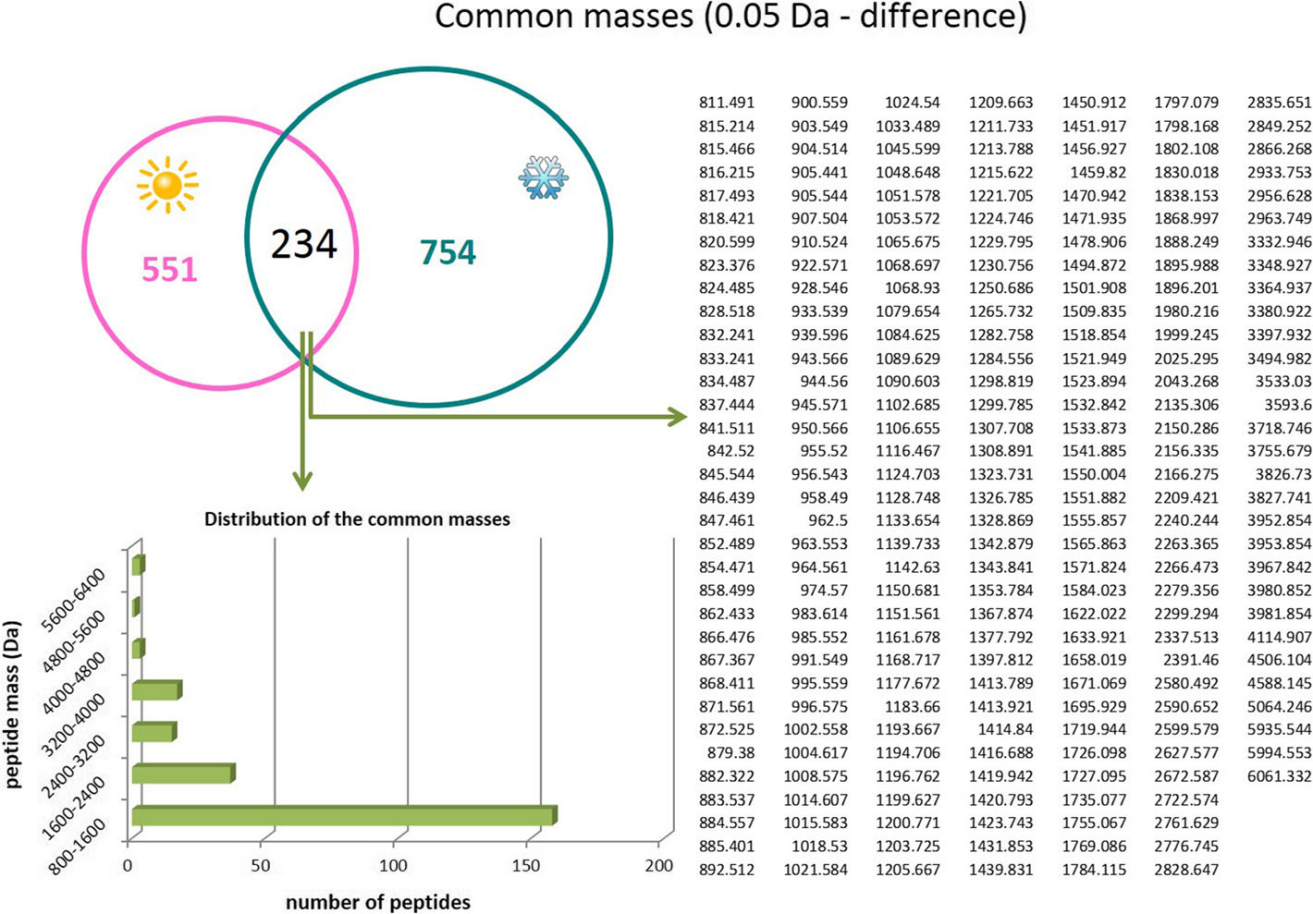
Ant venom peptide comparison. Venn diagram showing the unique and common venom peptides (intersection) collected during summer (**pink**) and winter (**blue turquoise**). A list of the common masses is shown on the right and the mass distribution of those peptides is shown on the left

Both the mass lists were compared and masses matching within 0.05 Da were considered identical. Due to the high resolution of the mass spectrometer used, a low mass difference (0.05 Da) could be fixed to compare both conditions. The analysis showed that 234 peptides (15%) were “identical” for both venoms as shown in Fig. 3. The mass distribution of the identical peptides (Fig. 2) follow the same distribution of the peptides of each venom (Fig. 3), with most peptides grouped in the 800–1600 Da range. The common masses are shown in Fig. 3.

**Fig. 4:**
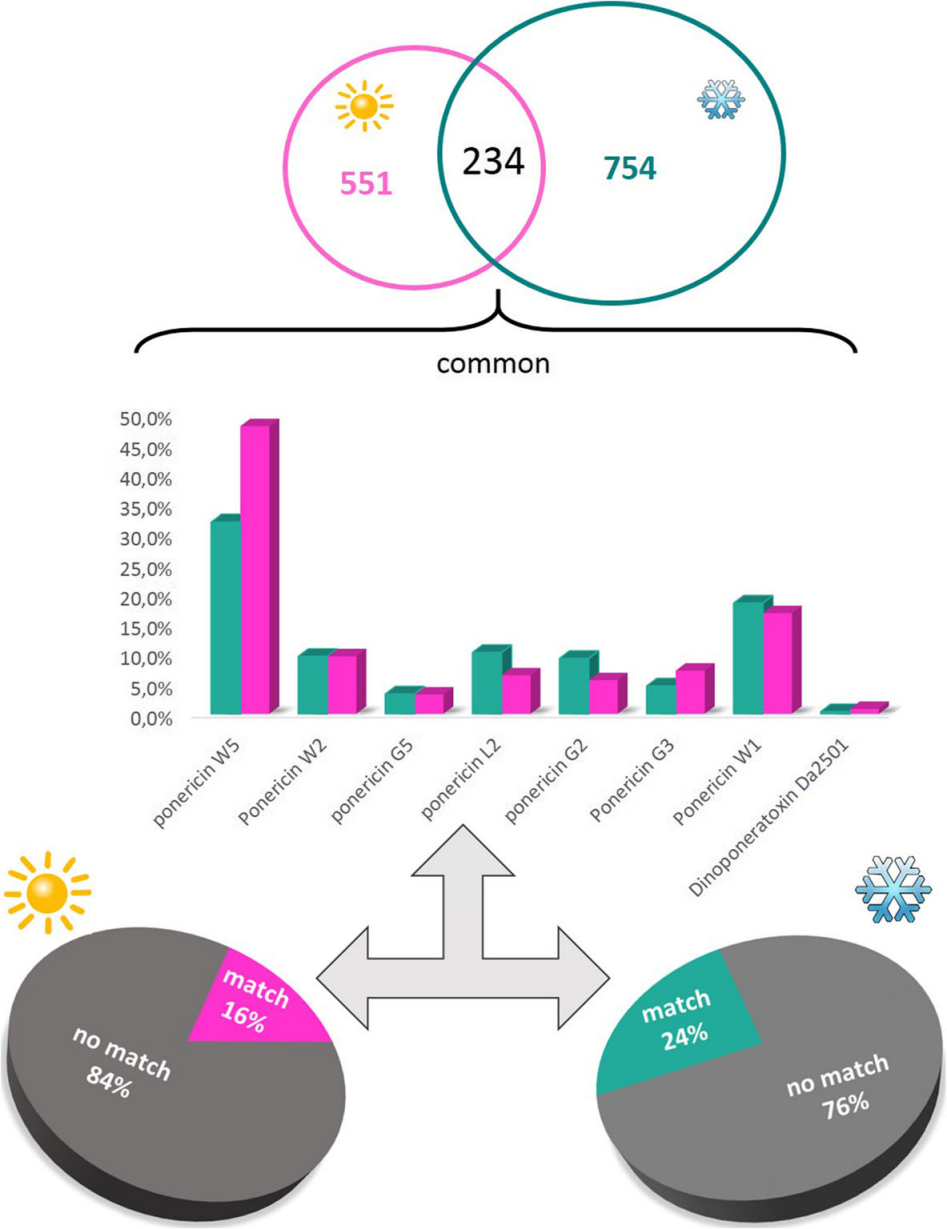
Analysis of the results obtained by PEAKS using the animal toxin database. The raw data obtained by high resolution nano-LC-ESI-MS/MS of venoms extracted during summer (**pink**) and winter (**blue turquoise**) were upload in PEAKS 7. Most of the tags obtained after the automated de novo sequencing did not have any match against the database used (pie charts). The common peptides identified were mainly from the ponericin family (bar graph). The bar graph shows the abundance of the matched peptides for each condition. Just matched peptides that were common for both conditions are shown

The peptides obtained by high resolution nano-LC-ESI-MS/MS were de novo sequenced generating high quality sequence tags that were used by PEAKS DB and Spider algorithm dedicated to the searches into specifics databases such as the hymenoptera and animal toxins database. We have considered as an accurate identification just the peptides that presented more than 40% coverage. The animal toxins database showed the best matches and therefore was chosen for the analysis. A table including all matches (coverage > 40%) is available in Additional file 1. Among the results obtained, we highlight the ponericins, a well-known antimicrobial peptide family (Fig. 4, bar graph), which were common in both venom conditions. Besides that, the common peptides also matched with dinoponeratoxins (Fig. 4, bar graph). In addition, we emphasize the percentage that did not have any match with the database used (76 and 84%), as shown in Fig. 4 (pie chart).

### Venom comparisons – ground-dwelling × arboreal

**Fig. 5:**
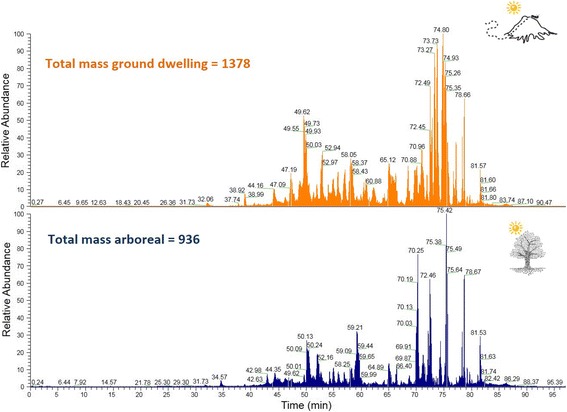
Total ion chromatogram (TIC) of the crude venom from ground-dwelling (**orange**) and arboreal (**blue**) ants

**Fig. 6:**
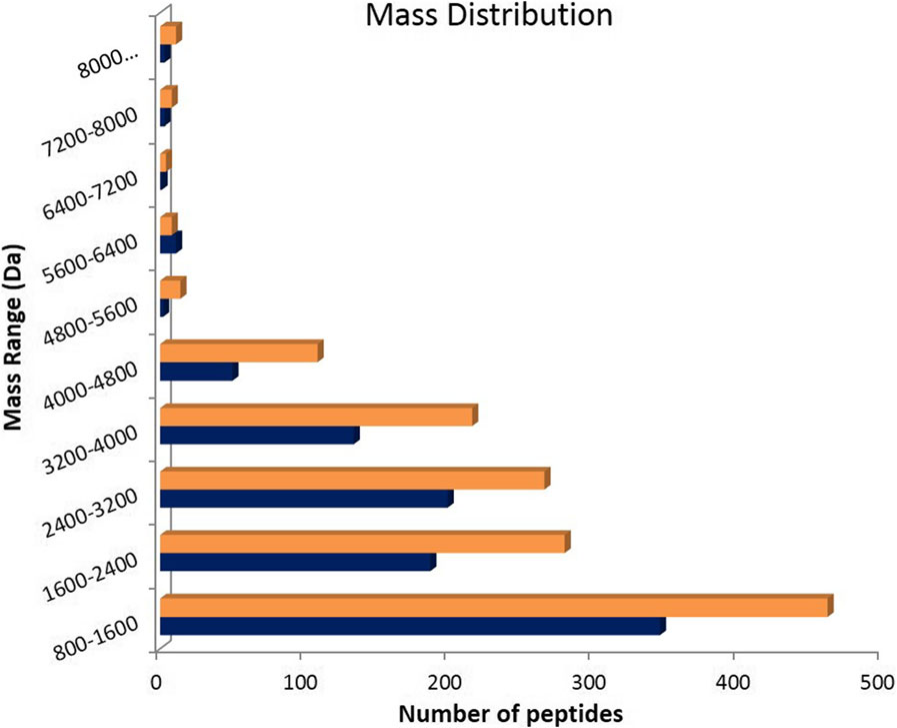
Mass distribution of the venom peptides extracted from arboreal (**blue**) and ground-dwelling (**orange**) ants

**Fig. 7:**
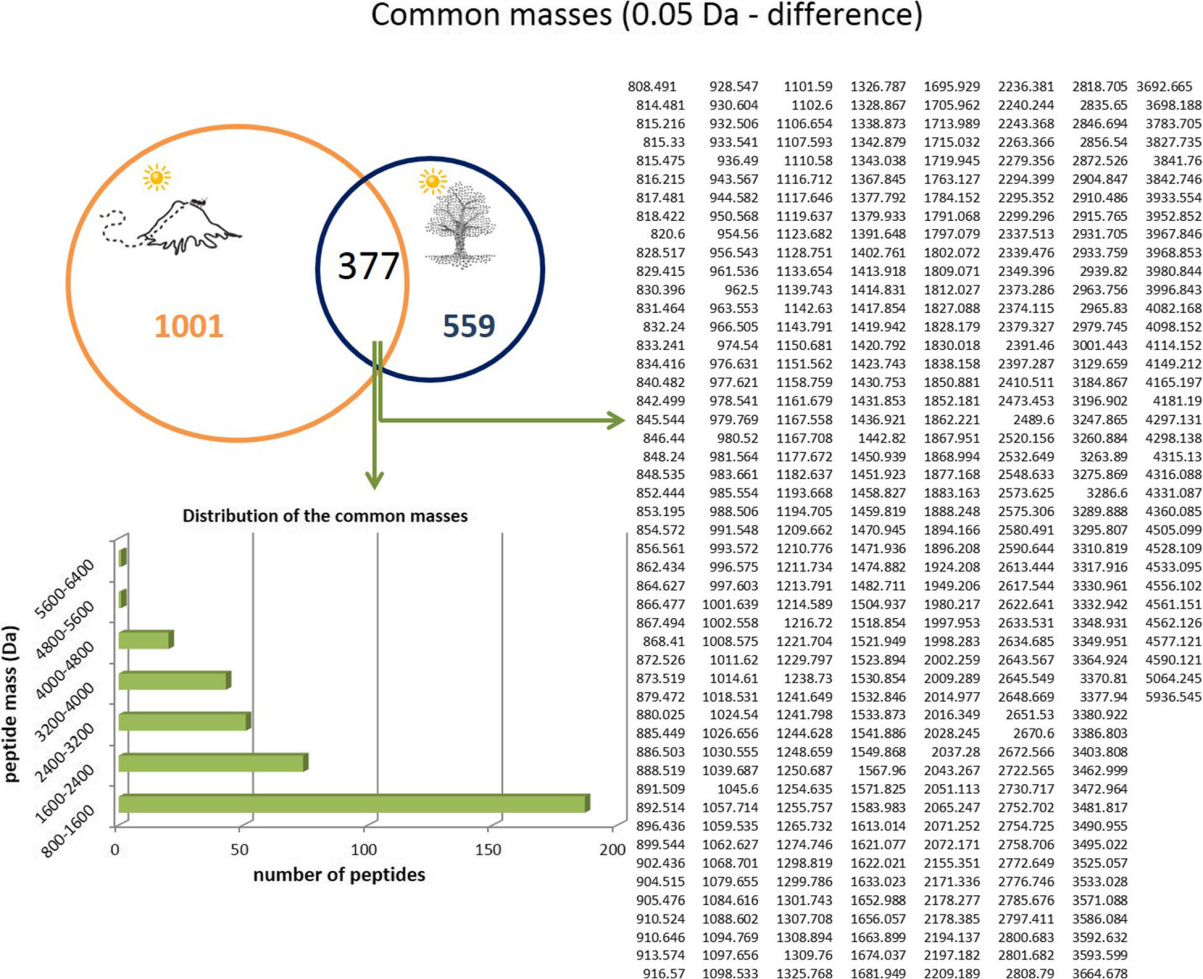
Venom peptide comparison. Venn diagram showing the unique and common venom peptides (intersection) extracted from ground-dwelling (**orange**) and arboreal ants (**blue**). The mass list of the common masses is shown on the right and the mass distribution of those peptides is shown on the left

The venom collected from arboreal and terrestrial nests were compared (venoms were extracted during summer). The same experiments performed for the venoms described in the previous comparison were adopted for this comparison. The total ion chromatogram (Fig. 5) demonstrates that the venom from terrestrial nest ants is more complex than the venom of ants that live in tree trunks. The venom of arboreal ants exhibited 936 peptides in its composition, while terrestrial ants presented 1378 peptides in their venom. The distribution of the molecular masses is similar for both venoms, as shown in Fig. 6. Arboreal and ground-dwelling ants presented 377 (19%) peptides in common (Fig. 7). The common mass values are illustrated in Fig. 7.

**Fig. 8:**
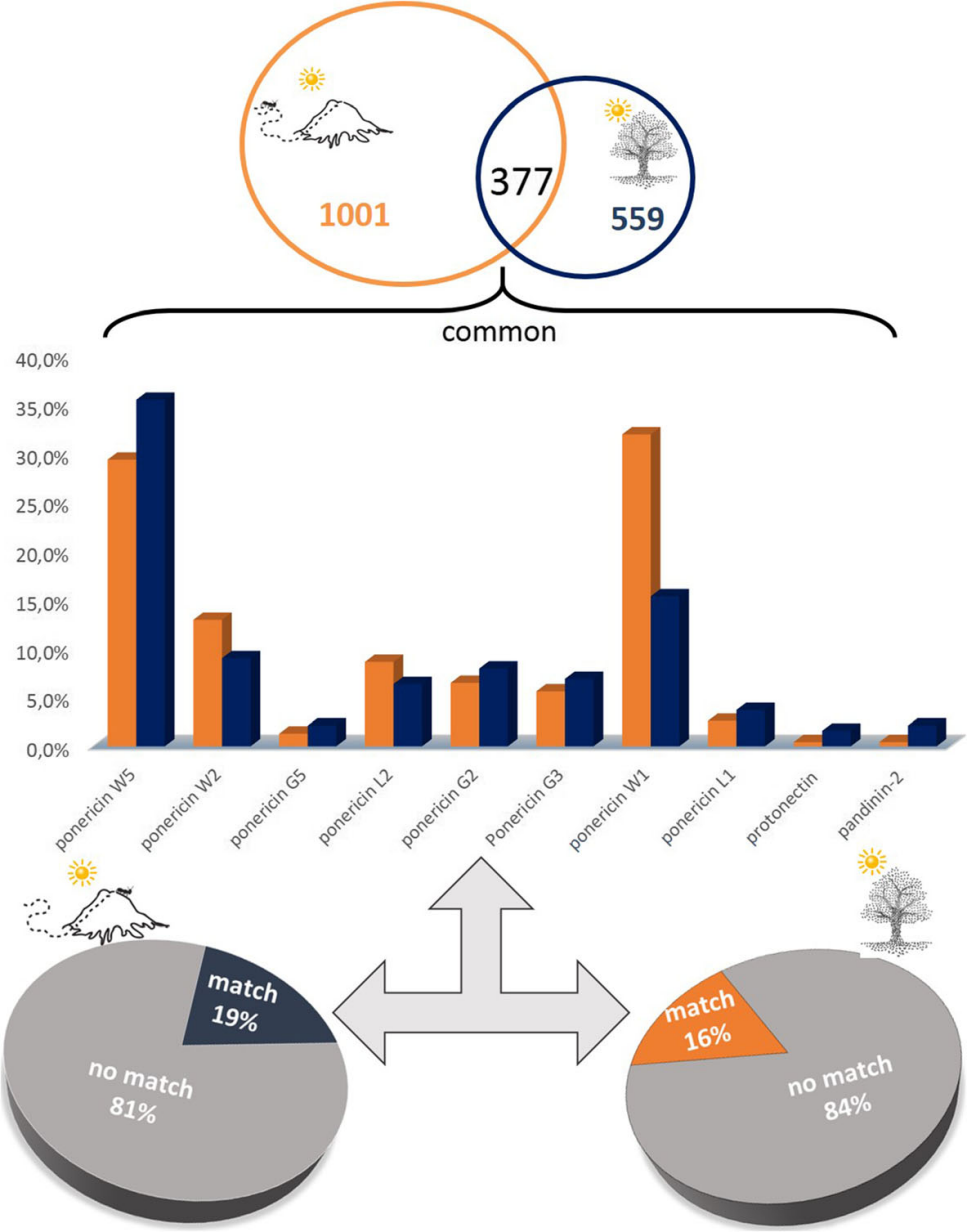
Analysis of the results obtained by PEAKS using the animal toxin database. The raw data obtained by high resolution nano-LC-ESI-MS/MS of venoms extracted from ground-dwelling (**orange**) and arboreal ants (**blue**) were uploaded in PEAKS 7. Most of the tags obtained after the automated de novo sequencing did not show any match against the database used (pie charts). The common peptides identified, shown on histogram graph, were mainly from ponericin family (bar graph). The bar graph shows the abundance of the matched peptides for each condition. Just matched peptides that were common for both conditions are shown

The peptides obtained by high resolution nano-LC-ESI-MS/MS were de novo sequenced generating high quality sequence tags which were used by PEAKS DB and Spider algorithm following the same parameters used for the summer and winter analysis. A table including all matches (coverage > 40%) is available in the Additional file 2. Among the results obtained, we highlight again the ponericins (Fig. 8) that were common in both venom conditions. In addition, the common peptides also matched with pandinin-2 and protonectin, as shown in Fig. 8 (bar graphs). Once more, we emphasize the percentage of peptides that did not have any match within the database used (81 and 84%).

## Discussion

### Venom comparison

Advancements in mass spectrometry that use soft ionization techniques such as matrix assisted laser desorption ionization (MALDI) and electrospray, along with the development of proteomic and peptidomic strategies have turned the characterization of animal venoms more complete and easier to embrace by fundamental research [1, 15, 16]. Venom from different species of animals has been explored using this technique, including species previously left aside, such as ants [1, 2]. Despite the limited research concerning ant venom, a considerable number of studies unveiling the venom complexity has already been published using a proteomic/peptidomic approach [2, 9, 17, 18]. In the present work, four peptidomes of the venom of *N. villosa* were decoded using a high-resolution mass spectrometry coupled with nano-chromatography. The peptidomes were constructed in order to compare and elucidate the climatic and habitat influences in the composition of the venom.

Thus, ant venoms collected in summer and winter were compared revealing considerable differences. The results have shown that only 15% of the peptides are shared between the two seasonally different conditions, revealing a lavish plasticity. Ferreira Junior and et al. [19] have already reported that melittin and phopholipases A_2_, from bee venom, differ according to climatic and seasonal factors. This seasonal variation was also described for the antigen 5-like gene, which is expressed by the bee venom gland in winter but not during summer [20]. In ants, as well as all the Hymenoptera, only females are venomous, eliminating venom variability related to gender. The ontogenic variation could also be excluded since both old (foraging workers) and young (living inside the nest) specimens were collected. In addition, the ants were collected in the same geographic region, thus excluding the influence of this variable on our experiments.

Danneels and et al. [21] have compared the venom composition of winter and summer bees, describing differences related to the fact that bees face different predators and intruders during the two seasons. Mind that ant venom, as well as other aculeate species, has not only an offensive function for prey capture, but also a defensive role against possible attackers, including defense against antimicrobials by preventing infections within their colonies [2, 5, 10, 22]. As well as predators and intruders may change, the type of available prey may oscillate along the year in response to climatic and seasonal fluctuations, putting diet and defense on the spotlight of venom variation.

It has been demonstrated that some social insects display seasonal shifts in foraging behavior [23]. The authors demonstrated a link between seasonal food collection behavior and nutrient regulation strategies, suggesting that season-specific nutrient regulation strategies may be an adaptation of ants (amongst other animals) to meet current and long-term nutrient demands when nutrient-rich food is abundant (spring and summer) and to conserve energy when food is less abundant [23]. Consistently, a marked increase of foraging activity in a warm and wet season by ponerine ants was already reported in savanna and forest ecosystems [24–26]. Although *Neoponera* genus consists of polyphagous ants, insects constitute the major food source and, thus, their venom must be empowered to immobilize and kill these preys [10].

In the current work, ants were captured in the *cerrado* ecoregion characterized by a tropical climate with two distinct seasons: dry winter (from May to September) and rainy summer (from October to April) [27]. During summer, hot and rainy, the ants are more active, foraging and storing food before the beginning of the winter (which is still warm, but dry) and, thus, the ants would be using more often their venom. From this perspective, the lower number of peptides in the venom collected in summer (551 peptides) when compared to that collected in winter (754 peptides) is explained. Therefore, we support the idea that climate changes, that influence prey availability and predator presence, have an impact on the expression of peptides that compose *N. villosa* venom. It is worth mentioning that it is not yet possible to state whether this variability is due to a plasticity in gene expression in response to environmental changes – such as the abstinence of certain types of breed or presence of certain microbial strains in the colony – or whether this change in venom composition is the result of an evolutional adaptation to habitats with marked seasonality.

When comparing the different types of nesting that these ants can adopt (ground-dwelling or arboreal), remarkable differences were identified. Among the peptides composing the venom of ground-dwelling and arboreal ants, only 377 (19%) are common for both conditions. Orivel et al. [10] verified that the venom of the *Neoponera* ants that adopt different types of nesting presented similar biological activities, but with different efficacies. The authors stated that the paralysis and the lethality effect of arboreal ant venoms are significantly different when compared to the venom of ground-dwelling ants. This variation in the potency of venom activity represents an adaptation to arboreal life, since the possibility of prey to escape in this environment is greater when compared to terrestrial life [10]. Recently, it was reported that the venom toxicity of three *Pseudomyrmex* ant species, which have different nesting habits, did not vary, but their compositions were remarkably different [28]. In the present work, the peptides present in the ground-dwelling venom are more numerous than the arboreal one; however, we cannot stress anything yet concerning their efficiency.

Intraspecific variations of venoms have already been reported for several groups of animals such as snakes, scorpions, spiders and some hymenopterans. Such variability is often related to geographic distribution, age, gender and diet [9, 29, 30]. For some species, such as snakes, to elucidate intraspecific venom variation is of the utmost importance to understand the envenoming process and produce more efficient antivenom [31]. Concerning species with less medical importance, the exploration of these variations represents a golden key to unveil novel bioactive compounds and may shed light on venom evolution.

The raw data from these four peptidomes were entered into the PEAKS software for database search and automated de novo sequencing. The results obtained indicate that some of the common peptides for the four investigated situations correspond to ponericins (Figs. 4 and 8). The ponericins are a group of 27 peptides isolated from the venom of *N. goeldi*, *N. apicalis* and *N. inversa* ants. These peptides adopt amphipathic structures and have shown hemolytic, antibacterial (both gram-positive and gram-negative), antifungal and insecticidal activity [8]. These activities are important to prevent the spread of microbial pathogens inside the colony (by means of infected food, for instance) and to subdue prey, as these ants can feed on small insects [5, 8]. Since its presence has been identified in all conditions of the studied venoms, it is suggested that these peptides are fundamental for the survival of ants and the colony.

A very interesting fact on the analyses caught our eyes: the presence of not only the complete peptide corresponding to a ponericin subtype, but also smaller fragments of the same antimicrobial peptide (Table 1). This observation occurred not only in the ponericins W5, as Table 1 illustrates, but also in other ponericins present in the venom. This phenomenon may indicate: degradation of the samples, or that this class of antimicrobial peptides might undergo enzymatic cleavages. This peptide processing seems to occur at both extremities of the peptide, suggesting the action of carboxypeptidases, aminopeptidases and/or endopeptidases (Table 1). This extensive proteolysis was observed only in ponericins and not in the other peptides identified in our work, suggesting an enzymatic preference to this peptide subfamily. In this way, it appears to be implausible that the peptide proteolysis observed is caused by sample degradation but it is, indeed, the result of post-translational modifications.

**Table 1:** Full sequence of ponericin W5 (P82427) and its fragments. The full peptide corresponding to ponericin W5 and its fragments were identified in the venom of *N. villosa*. Isoforms of the full peptide and its fragments were also identified

Sequence	Condition	Mw (Da)	Peptide processing/Mutations
FWGALIKGAAKLIPSVVGLFKKKQ	Ponericin W5	2598.57	–
FWGALIKGAAKLIPSVVGLFKKKQ	Nv1; Nv2; Nv3; Nv4	2598.57	–
FWGALIKGAAKLIPSVVGLFKKK	Nv1;Nv3; Nv4	2470.51	Carboxypeptidase
FWGALIKGAAKLIPSVVGLFKK	Nv1	2342.42	Carboxypeptidase
FWGALIKGAAKLIPSVVGLF	Nv4	2086.23	Carboxypeptidase
FWGALIKGAAKLIPSVVGL	Nv2;Nv4	1939.16	Carboxypeptidase
FWGALIKGAAKLIPSVVG	Nv1; Nv3; Nv4	1826.08	Carboxypeptidase
FWGALIKGAAKLIPSVV	Nv1	1769.06	Carboxypeptidase
ALIKGAAKLIPSVVGLFKK	Nv4	1952.25	Endopeptidase
ALIKGAAKLIPSVVGLF	Nv1	1696.06	Endopeptidase
ALIKGAAKLIPSVVG	Nv4	1548.99	Endopeptidase
GAAKLIPSVVGLFKK	Nv1	1526.95	Endopeptidase
GAAKLIPSVVGLF	Nv4	1270.76	Endopeptidase
AAKLIPSVVGLF	Nv1;	1213.74	Endopeptidase
IPSVVGLFKK	Nv4	1342.83	Endopeptidase
PSVVGLFKKK	Nv3;	1101.69	Endopeptidase
IPSVVGLFK	Nv4	958.58	Endopeptidase
WGALIKGAAKLIPSVVGLFKKKQ	Nv1;	2451.50	Aminopeptidase
GALIKGAAKLIPSVVGLFKKKQ	Nv4	2265.42	Aminopeptidase
ALIKGAAKLIPSVVGLFKKKQ	Nv1;Nv4	2208.40	Aminopeptidase
IKGAAKLIPSVVGLFKKKQ	Nv1; Nv4	2024.28	Aminopeptidase
GAAKLIPSVVGLFKKKQ	Nv1; Nv2;Nv4	1783.10	Aminopeptidase
AAKLIPSVVGLFKKKQ	Nv1;Nv2; Nv4	1726.08	Aminopeptidase
PSVVGLFKKKQ	Nv1; Nv2; Nv4	1229.74	Aminopeptidase
FWGALIKGAAKLIPSVVG**M**FKKKQ	Nv1; Nv3; Nv4	2616.53	(Leu19Met)
FWGALIKGAAKLIPSVVG**M**	Nv3	1957.12	(Leu19Met)/carboxypeptidase
GALIKGAAKLIPSVVG**M**FKKKQ	Nv3	2283.38	(Leu19Met)/aminopeptidase
ALIKGAAKLIPSVVG**M**FKKKQ	Nv3	2226.36	(Leu19Met)/aminopeptidase
ALIKGAAKLIPSVVG**M**	Nv3	1566.95	(Leu19Met)/endopeptidase
GAAKLIPSVVG**M**FKKKQ	Nv1;Nv3	1801.06	(Leu19Met)/endopeptidase
FW^*^GALIKGAAKLIPSVVGLFKKKQ	Nv2; Nv4	2614.57	*Oxidation
FW^*^GALIKGAAKLIPSVVGL	Nv2	1955.16	*Oxidation/carboxypeptidase
FW^*^GALIKGAAK	Nv3	1176.66	*Oxidation/carboxypeptidase

Nv1: winter; Nv2: summer; Nv3 ground-dwelling ants; Nv4: arboreal ants

Bold letter indicates a mutation

* Indicates an oxidation

Toxin proteolysis was previously described and was related to the increase of the structural and molecular diversity of the venom protein repertoire [32]. Thus, with a single gene product cleaved in different positions, several other peptides with different targets and modes of action are produced, therefore generating an immense molecular repertoire with low energy costs [32]. Considering this molecular diversity enrichment of the venom composition by proteolysis, the quantitative and qualitative differences observed among the conditions compared could be related to the presence of several and diverse peptide fragments, yet belonging to the same venom peptide subfamily. In other words, each venom condition may present a similar global venom composition with the presence of the same peptide subfamilies (i.e. ponericins W5, as shown in Table 1), but differential fragmentation of those peptides produce a particular diversity. In addition, isoforms of the full peptide and its fragments were also detected. As shown in Table 1, a mutation (Leu19Met) of a ponericin W5 was identified both on the full peptide and its fragments. The mutation was found in more than one venom condition (Table 1) and highlights the chemical diversity that these bio-libraries may hold.

In addition to ponericins, we have identified the presence of peptides related to protopolybiakin-I, pandinin-2, dinoponeratoxin Da2501 and protonectin. Protopolybiakinin-I was isolated from *Protopolybia exigua*, a social wasp, and it was described to cause constriction of isolated rat ileum muscles and degranulation of mast cells. This peptide also causes analgesic effects due to the direct activation of B2-receptors [33]. The peptide pandinin-2, identified from the venom of the scorpion *Pandinus imperator*, disrupts cell membranes through formation of pores. This peptide has strong antimicrobial activity against gram-positive bacteria and increases the efficacy of antibiotics when tested against *E. coli*, by facilitating their penetration into the bacteria. The peptide still holds antifungal and hemolytic activity [34]. Dinoponeratoxins were described by Johnson et al. [35] who related these peptides to antimicrobial ones. In fact, dinoponeratoxin Da2501 was described as a full sequence that was fragmented to a smaller peptide (dinoponeratoxin Da 1585). The small fragment shares homology with antimicrobial peptides found in frogs while the full fragment (Da 2501) shares homology with ponericins [35]. Protonectin was first isolated from the venom of the social wasp *Protonectarina sylveirae* and later identified in other wasp species. This peptide exhibits a potent antimicrobial activity, including against multidrug-resistant strains [36]. All these identified sequences were related to antimicrobial peptides, reinforcing the idea that the venom of this ant is a rich source of such biocompounds.

## Conclusions

The present study comprises the first peptidomic investigation and comparison of the venom from the neotropical ant *Neoponera villosa*. It was demonstrated that substantial differences exist among the venoms of *N. villosa* ants extracted in different seasons and from different nest habitats. The venom composition is affected by climate changes that influence prey availability and predator presence. Part of the peptides matched to ponericins, a well-known antimicrobial peptide family. Additionally, small peptides fragments related to ponericins were also identified, suggesting that this class of antimicrobial peptide might undergo enzymatic cleavages. The presence of those fragments may increase the molecular diversity of the venom. Besides those ponericins, most of the peptides did not have any match to other peptides present on the searched databases indicating that this venom is a treasure trove of novel biocompounds. Definitely, this peptidomic-based research revealed that ant venom is a complex cocktail of bioactive compounds and a rich source of antimicrobial peptides.

## Additional files

Additional file 1: Peptide assignment of *N. villosa* venom extracted during winter and summer identified by nano-LC-ESI-MS/MS. (DOCX 13 kb)

Additional file 2: Peptide assignment of *N. villosa* venom extracted from arboreal and ground-dwelling ants identified by nano-LC-ESI-MS/MS. (DOCX 13 kb)

## Abbreviations

ACN: Acetonitrile
AGC: Automatic gain control
ALC: Average local confidence
DDA: Data dependent acquisition
FA: Formic acid
FDR: False discovery rate
HCD: High-energy dissociation
MALDI: Matrix assisted laser desorption ionization
MS: Mass spectrometry
Nano-LC-ESI-MS/MS: Nanoscale liquid chromatography coupled to electrospray tandem mass spectrometry
TIC: Total ion chromatogram
