# Supplementary material for: Peptidomic investigation of Neoponera villosa venom by high-resolution mass spectrometry: seasonal and nesting habitat variations
Source: J Venom Anim Toxins Incl Trop Dis. 2018 Feb 17;24:6. doi: 10.1186/s40409-018-0141-3 (PMC5816382; doi:10.1186/s40409-018-0141-3)
Supplement: Supplementary file 1 — Peptide assignment of N. villosa venom extracted during winter and summer identified by nano-LC-ESI-MS/MS. (DOCX 13 kb) [file 40409_2018_141_MOESM1_ESM.docx]

| **Protein/source** | **Accession code** | **Coverage/number of peptides/**  **score (-10 lgP)**  **Winter** | **Coverage/number of peptides/**  **score (-10 lgP)**  **Summer** |
| --- | --- | --- | --- |
| Ponericin-L2 | \|P82422\| | 100%/31 peptides/205.24 | 100%/8 peptides/126.19 |
| Ponericin-G3 | \|P82416\| | 100%/14 peptides/162.32 | 100%/9 peptides/151.13 |
| Ponericin-W5 | \|P82427\| | 100%/31 peptides/205.24 | 100% /60 peptides/314.51 |
| Ponericin-W1 | \|P82423\| | 100%/56 peptides/274.09 | 100%/21 peptides/194.08 |
| Ponericin-W2 | \|P82424\| | 84%/29 peptides/207.43 | 100%/97 peptides/391.56 |
| Ponericin-G2 | \|P82428\| | 100%/28 peptides/266.91 | 97%/7 peptides/144.41 |
| Ponericin-G5 | \|P82418\| | 73%/10 peptides/136.61 | 73%/4 peptides/121.72 |
| Ponericin-W6 | \|P82428\| | 100%/35 peptides/277.21 | – |
| Ponericin-L1 | \|P82421\| | – | 71%/4 peptides/90.02 |
| Dinoponeratoxin Da-2501 | \|P0CF01\| | 43%/1 peptide/42.86 | 43%/1 peptide/46.27 |
| Eumenine mastoparan-AF | \|P0C022\| | 64%/1 peptide/29.73 | – |
| PANIM Pandinin-2 | \|P83240\| | 46%/1 peptide/82.62 | – |
