# Supplementary material for: Peptidomic investigation of Neoponera villosa venom by high-resolution mass spectrometry: seasonal and nesting habitat variations
Source: J Venom Anim Toxins Incl Trop Dis. 2018 Feb 17;24:6. doi: 10.1186/s40409-018-0141-3 (PMC5816382; doi:10.1186/s40409-018-0141-3)
Supplement: Supplementary file 2 — Peptide assignment of N. villosa venom extracted from arboreal and ground-dwelling ants identified by nano-LC-ESI-MS/MS. (DOCX 13 kb) [file 40409_2018_141_MOESM2_ESM.docx]

| **Protein/source** | **Accession code** | **Coverage/number of peptides/**  **score (-10 lgP)**  **Ground-dwelling** | **Coverage/number of peptides/**  **score (-10 lgP)**  **Arboreal** |
| --- | --- | --- | --- |
| Ponericin-L2 | \|P82422\| | 100%/20 peptides/215.33 | 100%/12 peptides/178.29 |
| Ponericin-G3 | \|P82416\| | 100%/13 peptides/185.70 | 100%/13 peptides/180.65 |
| Ponericin-W5 | \|P82427\| | 100%/68 peptides/363.14 | 100%/67 peptides/393.43 |
| Ponericin-W1 | \|P82423\| | 100%/74 peptides/296.53 | 100%/29 peptides/235.13 |
| Ponericin-W2 | \|P82424\| | 96%/30 peptides/2010.58 | 88%/17peptides/176.93 |
| Ponericin-G2 | \|P82428\| | 97%/15 peptides/279.10 | 97%/15 peptides/218.48 |
| Ponericin-G5 | \|P82418\| | 80%/3 peptides/92.01 | 73%/4 peptides/138.88 |
| Ponericin-W6 | \|P82428\| | – | 100%/13 peptides/202.40 |
| Ponericin-L1 | \|P82421\| | 100%/6 peptides/139.05 | 100%/7 peptides/120.42 |
| Dinoponeratoxin Da-2501 | \|P0CF01\| | – | 65%/4 peptide/111.05 |
| Eumenine mastoparan-AF | \|P0C022\| | 64%/1 peptide/64.62 | – |
| PANIM Pandinin-2 | \|P83240\| | 46%/1 peptide/70.36 | 100%/4 peptides/107.29 |
| Protopolybiakinin-1 | \|P0DM70\| | – | 52%/2 peptides/94.68 |
| Protonectin | \|P69437\| | 100%/1 peptide/50.28 | 100%/1 peptide/99.81 |
